# Preparation of Lake Pigment from Calcium Carbonate and Cyanidin-3-O-Glucoside: Structural Characterization and Formation Mechanism

**DOI:** 10.3390/foods15132409

**Published:** 2026-07-07

**Authors:** Yifen Fu, Jiaqi Cui, Jiaxuan Dong, Chengtao Wang, Dongdong Yuan

**Affiliations:** Beijing Engineering and Technology Research Center of Food Additives, School of Food and Health, Beijing Technology and Business University, Beijing 100048, China; 18834695836@163.com (Y.F.);

**Keywords:** Cyanidin-3-O-glucoside, CaCO_3_, stability improvement, lake pigment, adsorption mechanism

## Abstract

To explore potential strategies for improving the applicability of cyanidin-3-O-glucoside (C3G) and to avoid the health risks associated with the in vivo accumulation of aluminum by intake of traditional aluminum-based lake pigments, food-grade CaCO_3_ was used as a matrix to prepare two types of edible lake pigments, namely C3G-CaCO_3_ and MA-CaCO_3_, via coprecipitation method using purified cyanidin-3-O-glucoside (C3G) and non-purified mulberry anthocyanins (MA). The effect of pH on adsorption was systematically investigated, and various characterization methods were used to analyze the physicochemical properties and formation mechanism of lake pigments. The results showed that pH 9.5 was the optimal condition for CaCO_3_ to adsorb MA. The introduction of C3G altered the particle size, surface charge, and other characteristics of CaCO_3_ without changing its calcite crystal form. The adsorption of MA and C3G on the CaCO_3_ surface was multilayer physical adsorption, dominated by the Freundlich model. The isothermal adsorption results showed that CaCO_3_ exhibited a higher adsorption capacity for C3G than for MA at equivalent equilibrium concentrations, demonstrating C3G’s superior binding affinity. C3G primarily binds to calcium carbonate through surface adsorption, with possible partial diffusion of molecules into the matrix, without forming new chemical bonds, and slightly regulated the thermal stability of CaCO_3_. This study successfully constructed a lake pigment system based on CaCO_3_, systematically elucidated its adsorption behavior and structural characteristics toward anthocyanins, and provided a material foundation for the further application of this type of carrier in the food sector.

## 1. Introduction

Anthocyanins are widely distributed water-soluble natural pigments in nature [1], among which cyanidin-3-O-glucoside (C3G) is the most abundant anthocyanin in plants [2], with a molecular formula of C_21_H_21_O_11_. As a nontoxic and harmless natural coloring substance, C3G has been applied in the food, cosmetic, and nutraceutical industries [3]. In terms of physiological activities, existing literature has reported that C3G exhibits antioxidant [4] and anti-inflammatory effects [5]. In addition, some studies have also explored its potential intervention value in neurological disorders [6]. Since C3G possesses both coloring properties and multiple biological activities, it has become one of the compounds of great interest in the field of natural product research in recent years. However, C3G is chemically highly unstable and is susceptible to degradation during processing and storage due to various environmental factors such as pH, oxygen, light, and temperature [7]. Various encapsulation technologies for anthocyanins have been widely reported [8,9,10,11,12], primarily aiming to enclose pigments within the matrix to enhance stability and bioactivity. However, this interior loading inevitably reduces color visibility, undermining their role as colorants. Moreover, each approach has food-application drawbacks: organic matrices (biopolymers, proteins, polysaccharides, nanoparticles) exhibit poor powder properties, instability, and inherent coloration; copigmentation efficacy varies greatly with system composition and pH, with limited solid-food applicability; mineral systems have low loading capacity and cause dull coloration, poor flowability, and hygroscopic caking. In contrast, lake pigment technology anchors anthocyanins on the matrix surface, preserving coloration while ensuring stability. This study uses food-grade CaCO_3_ as the mineral carrier to effectively overcome these issues.

Aluminum hydroxide is a classic adsorption matrix for traditional edible lake pigments [13], with excellent adsorption performance. The prepared lake pigments also have the advantages of bright color, good stability, and strong tinting strength. However, its health risks have drawn growing concern. Aluminum accumulates in the body, with biotoxicity affecting the digestive and respiratory systems [14,15], and excessive intake may induce oxidative stress, inflammation, brain aging, and neurodegenerative disorders [16]. China’s GB 2760-2014 (and revisions) strictly regulates aluminum-containing additives, banning indigo and erythrosine aluminum lake pigments in children’s foods like puffed snacks, thereby driving industrial innovation.

Calcium carbonate, a common food additive, has a wide range of applications and serves as a calcium supplement, colorant, leavening agent, and antacid [17,18,19]. Although calcium carbonate cannot form a loose flocculent structure in solution, as aluminum hydroxide does, and thus cannot obtain a large specific surface area, it is still widely used as an adsorbent because of its flexible surface modifiability and significant surface charge [20]. Our research group has previously prepared an indigo carmine–calcium carbonate lake pigment [21], which was applied to various foods to enhance pigment stability. Subsequently, a monascus red–calcium carbonate lake pigment was developed [22], whose application in smoked sausage can effectively improve pigment performance and realize the substitution of nitrite [23]. Meanwhile, *β*-carotene–calcium carbonate lake pigment was successfully prepared, and its formation mechanism was studied [24].

Based on the above research background, this study builds upon previous work and proposes the application of CaCO_3_-based lake technology to a typical anthocyanin, cyanidin-3-O-glucoside (C3G) from mulberry MA. By employing a series of technologies, including scanning electron microscopy (SEM), X-ray diffraction (XRD), isothermal adsorption, Fourier-transform infrared spectroscopy (FTIR), Raman spectroscopy, thermogravimetric analysis (TGA) and differential scanning calorimetry (DSC), this study aimed to systematically elucidate the formation mechanism and structure–activity relationship of the C3G-CaCO_3_ lake pigment by clarifying the spatial distribution pattern of C3G within CaCO_3_, characterizing the intermolecular physical interactions, and revealing the regulatory effect of C3G on the thermal stability of CaCO_3_. The findings of this article not only further enrich the variety of calcium carbonate lakes, but also point the way for more in-depth theoretical research.

## 2. Materials and Methods

### 2.1. Chemicals

Mulberry anthocyanins (~25% *w*/*w*, containing C3G) were purchased from Shaanxi Baichuan Kangze Biotechnology Co., Ltd. (Xi’an, China), and the C3G standard (>98% *w*/*w*) was obtained from Shanghai Yuanye Biotechnology Co., Ltd. (Shanghai, China). All other chemical reagents, including calcium chloride (96% *w*/*w*), sodium carbonate (99.9% *w*/*w*), ethanol (99.7% *w*/*w*), sodium hydroxide (>98% *w*/*w*), and hydrochloric acid (~37% *w*/*w*), were of analytical grade and purchased from Sinopharm Chemical Reagent Co., Ltd. (Shanghai, China). Deionized water (~18.25 MΩ·cm) used for solution preparation in this study was obtained from the laboratory water purification system (HYP-QX-UP, Beijing, China).

### 2.2. Preparation of MA-CaCO_3_ Lake Pigments

Preparation of MA-CaCO_3_ lake pigment: Deionized water (25.0 g) was weighed into a 50.0 mL centrifuge tube, and anhydrous calcium chloride (2.1 g) was added and dissolved by shaking for later use. Sodium carbonate (2.0 g) was added to deionized water (200.0 g) and completely dissolved by magnetic stirring. MA (500.0 mg) was dissolved in the above sodium carbonate solution, and the pH was adjusted to 9.5 with 0.1 mol/L HCl/NaOH. The previously prepared calcium chloride solution was then mixed into the pH-adjusted mixed solution of sodium carbonate and MA with continuous magnetic stirring. Approximately 1.89 g of CaCO_3_ was formed (theoretical yield), and after precipitation was complete, the final pH of the reaction system was measured to be 7.8. The mixture was continuously stirred for 30 min to allow sufficient adsorption of MA onto the calcium carbonate precipitate, and then centrifuged (8000 rpm, 10 min) to obtain the supernatant containing unadsorbed MA. The supernatant was discarded to obtain the precipitate, which was dried in a vacuum drying oven at 45 °C for 12 h.

### 2.3. Adsorption Curves of MA-CaCO_3_ Lake Pigment via the Coprecipitation Method

Anhydrous sodium carbonate powder (4.0 g) was weighed and dissolved in 400.0 g of deionized water, followed by the addition of 1.0 g of MA. The mixture was thoroughly stirred with a magnetic stirrer to achieve complete dissolution. The resulting MA–sodium carbonate mixture was adjusted to specific pH (8.0, 8.5, 9.0, 9.5, 10.0, 11.0). Separately, 4.2 g of anhydrous calcium chloride powder was weighed and dissolved in 50.0 g of deionized water. After complete dissolution, the calcium chloride solution was mixed with the MA–sodium carbonate solution under stirring, and the timing was initiated simultaneously. At predetermined time intervals, 1.5 mL of the mixture was withdrawn using a syringe, and then filtered through a 0.22 μm aqueous filter membrane to obtain a clear filtrate. The absorbance of C3G in the filtrate was measured at the characteristic wavelength of 570 nm via ultraviolet spectrophotometry [25]. The amount of pigment adsorbed by CaCO_3_ at each time point was calculated and denoted as q_t_ (mg/g). The qt curves as a function of time were plotted accordingly. The adsorption data presented here have been processed by subtracting the corresponding blank controls (i.e., pigment solutions without CaCO_3_ at the same pH values and time points).

### 2.4. Isolation and Purification of C3G and Preparation of C3G-CaCO_3_ Lake Pigment

A commercial mulberry anthocyanin (MA) sample was separated and purified by an Agilent PrepStar SD-1 preparative liquid chromatography system equipped with an ultraviolet detector and a C18 chromatographic column. Aqueous formic acid–acetonitrile was used as the mobile phase, and the target peak corresponding to cyanidin-3-O-glucoside (C3G) was captured at a detection wavelength of 530 nm. The target fractions were selectively collected by an Agilent 440 automatic fraction collector. After combining the fractions, the organic solvents were removed by vacuum rotary evaporation at 40 ± 1 °C. The concentrated solution was pre-frozen at −80 °C and then transferred to a −80 °C freeze dryer for lyophilization for 50 h, finally obtaining high-purity C3G solid powder. The structure and purity of the extract were subsequently analyzed. Equal amounts of the extract and the C3G standard were dissolved to prepare sample solutions at the same concentration. The samples were then analyzed by high-performance liquid chromatography (HPLC) to obtain their chromatograms, and further structural characterization was performed using mass spectrometry (MS). The results confirmed that the major component of the extract was C3G, with a purity exceeding 90% (confirmed by HPLC and MS analyses in Figures S1 and S2). The preparation of C3G-CaCO_3_ lake pigment was carried out according to the method described in Section 2.2.

### 2.5. Characterization of Lake Pigments

Zeta potential and particle size: The average particle size and Zeta potential of the samples were determined using a Zetasizer Nano ZS90 dynamic light scattering instrument (Malvern Panalytical, Malvern, UK). Prior to measurement, the samples were diluted with deionized water (pH 6.8 ± 0.2) to a final concentration of 0.5 mg/mL, vortex-mixed, and then allowed to stand at 25 °C for 5 min. For particle size measurement, the dispersant refractive index was set to 1.330, with an equilibration time of 120 s. Each sample was measured in triplicate, and the results were expressed as mean ± standard deviation. Zeta potential measurements were performed using a capillary cell at a constant temperature of 25 °C, with each sample measured in triplicate, and the results were expressed as mean ± standard deviation.

BET specific surface area: Specific surface area is an important parameter that reflects the surface adsorption capacity and dispersion performance of lake pigment particles. The BET specific surface area of the lake pigment particles was determined by a NOVA4000e (Quantachrome, Boynton Beach, FL, USA) instrument with N_2_ adsorption under degassing conditions at 80 °C.

SEM: Scanning electron microscopy was used to observe the micromorphology and surface structure of the samples. The dried powder samples were subjected to vacuum gold sputtering treatment, and then the micromorphology of the three samples was observed by a scanning electron microscope (CX200PLUS COXEM, Daejeon, South Korea).

XRD: X-ray diffraction (XRD) was employed to analyze the crystal structures of CaCO_3_ and lacustrine pigments using an Empyrean diffractometer (Malvern Panalytical) with Cu Kα radiation (λ = 1.5406 Å) operating at 40 kV and 40 mA. The samples were scanned in the θ/2θ coupled continuous mode over a 2θ range of 5° to 90° at a scanning speed of 2°/min with a step size of 0.02°. The obtained diffraction data were processed using MDI Jade 6.5 software for phase identification and crystal structure analysis.

### 2.6. XPS and EDS Analysis

The lake pigment samples were vacuum-dried at 45 °C for 12 h and then ball-milled to obtain powder samples with uniform particle size for characterization analysis. Surface elemental analysis was performed by a Thermo Scientific ESCALAB Xi+ X-ray photoelectron spectrometer (Waltham, MA, USA) to detect the binding energies of the C 1s, O 1s, and Ca 2p orbitals. To ensure the reliability of the fitting results, the peak-fitting analysis of the C 1s high-resolution spectra was performed using the Peak Analyzer module in Origin software. During the fitting process, a mixed Gaussian–Lorentzian peak shape was employed in conjunction with Shirley background subtraction. All XPS spectra were corrected for charging effects by referencing the adventitious C 1s peak (C-C) to a binding energy of 284.8 eV. Meanwhile, XPS measurements were performed on representative samples prepared under identical conditions, with multiple replicate measurements conducted. The obtained spectra consistently exhibited stable and reproducible features.

Micromorphology characterization was completed by a Hitachi S-4800 cold field emission scanning electron microscope (Hitachi, Tokyo, Japan): The samples were brittle-fractured with liquid nitrogen and fixed on a conductive adhesive stage, and particles with typical cross-sectional morphology were selected for high-resolution imaging at an accelerating voltage of 5 kV. For the selected cross sections, the point scanning mode of EDS (step size 50 nm) was adopted to analyze the spatial distribution of characteristic X-ray signals of C-Kα (277 eV), O-Kα (525 eV), and Ca-Kα (3.69 keV).

### 2.7. Adsorption Isotherm Analysis

CaCl_2_ (0.52 g) and Na_2_CO_3_ (0.50 g) were weighed respectively and dissolved in 6.25 g and 50.00 g of deionized water in sequence to prepare stock solutions. The pigment (MA or C3G) was dissolved in the Na_2_CO_3_ solution to prepare a series of solutions with a concentration gradient of 4.44–13.33 mg/mL (4.44, 5.33, 6.22, 7.11, 8.00, 8.89, 9.30, 9.78, 10.22, 10.67, 11.11, 11.55, 12.44, 13.33 mg/mL, respectively), and the pH was adjusted to 9.50 ± 0.05 with dilute NaOH/HCl solution. The CaCl_2_ solution was uniformly injected into the above mixed system, and the reaction was carried out at room temperature with magnetic stirring (500 rpm) for 30 min. The adsorption system had essentially reached equilibrium after 30 min. The reaction solution (1.50 mL) was filtered through a 0.22 μm aqueous membrane, and the filtrate was collected in a quartz cuvette. The absorbance value was determined by a UV-Vis spectrophotometer (λ = 570 nm), and the free pigment concentration (C_t_, mg/L) was calculated by the standard curve. The adsorption capacity q_e_ (mg/g) of CaCO_3_ for the pigment was calculated according to Equation (1). The adsorption curve was fitted to the Freundlich and Langmuir models.

(1) $$q_{e}=\frac{\left(C_{t}-C_{e}\right)V}{M_{caco_{3}}}$$

(2) $$\mathrm{Langmuir}:\;\frac{C_{e}}{q_{e}}=\frac{1}{q_{max}\times K_{L}}+\frac{C_{e}}{q_{max}}$$

(3) $$\mathrm{Freundlich}:\;ln\left(q_{e}\right)=ln\left(K_{F}\right)+\frac{ln\left(C_{e}\right)}{n}$$

where q_e_ is the adsorption capacity of CaCO_3_ for the pigment (mg/g); C_e_ is the concentration of unadsorbed C3G in the solution after 30 min (mg/L); C_t_ is the initial concentration of C3G before adsorption; V is the volume of the reaction solution (0.056 L); M_CaCO3_ is the theoretically calculated mass of calcium carbonate generated in the reaction (g); in this experiment, this value was 0.47 g. q_max_ is the maximum value of q_e_ (mg/g); K_L_ is the adsorption equilibrium constant of the Langmuir model (L/mg); n is the Freundlich constant (representing adsorption intensity); K_F_ is the adsorption equilibrium constant of the Freundlich model (mg^1−1/n·^g^−1·^L^1/n^). Among them, the Langmuir model reflects the monolayer adsorption of adsorbate on a homogeneous surface, while the Freundlich model is suitable for describing the multilayer adsorption process on heterogeneous surfaces.

### 2.8. Fourier Transform Infrared Spectroscopy (FTIR) Analysis

FTIR analysis of the samples was performed by a Nicolet Nexus 410 Fourier transform infrared spectrometer (Thermo Fisher, Waltham, MA, USA). Dried sample powder (2.0 mg; vacuum-dried at 60 °C for 12 h) and spectroscopic-grade KBr (200 mg) were weighed, ground uniformly in an agate mortar, and pressed into pellets to prepare test sheets. The parameters were set as follows: scanning range of 4000–500 cm^−1^, resolution of 4 cm^−1^, and scan number of 32. The acquired raw spectra were subjected to baseline correction followed by peak area normalization to ensure the comparability of spectral intensities among different samples.

### 2.9. Raman Spectroscopy Analysis

Raman spectroscopy was conducted on a HORIBA LabRAM Odyssey confocal micro-Raman spectrometer (Kyoto, Japan) using a 532 nm excitation laser over a Raman shift range of 100–3000 cm^−1^. The samples were pressed onto glass slides before measurement. The acquisition parameters were laser power of 2 mW; 50× long-working-distance objective (working distance ≥ 10.6 mm); single acquisition time of 20 s with 3 accumulations; and 1800 gr/mm grating (spectral resolution better than 0.65 cm^−1^). All samples were measured under identical conditions to ensure comparability. Calibration was performed using a silicon wafer, and all spectra were background-subtracted.

### 2.10. Thermogravimetric Analysis (TGA) and Differential Scanning Calorimetry (DSC)

The mass change and thermodynamic parameters of the samples were monitored synchronously by a TGA-DSC instrument (TA SDT Q600, New Castle, DE, USA) in the range of 30–800 °C. The samples were placed in standard aluminum crucibles, sealed with perforated aluminum lids, and heated at a constant rate of 10 °C/min under a nitrogen protective atmosphere. This analytical method can be used to characterize the thermal decomposition behavior of pure CaCO_3_ and the CaCO_3_ present in the lake pigment.

### 2.11. Data Statistics and Analysis

Data processing and mapping were completed using Microsoft Excel 2021 and Origin 2018. All experiments were performed in triplicate as independent replicates (i.e., each replicate was independently carried out from the initial sample preparation through the entire experimental workflow), and the results were expressed as mean ± standard deviation (SD). Means were compared through one-way ANOVA test using IBM SPSS Statistics 26, while differences were considered significant when *p* < 0.05.

## 3. Results

### 3.1. Effect of pH on Adsorption Curves of MA-CaCO_3_ via Coprecipitation

**Figure 1:**
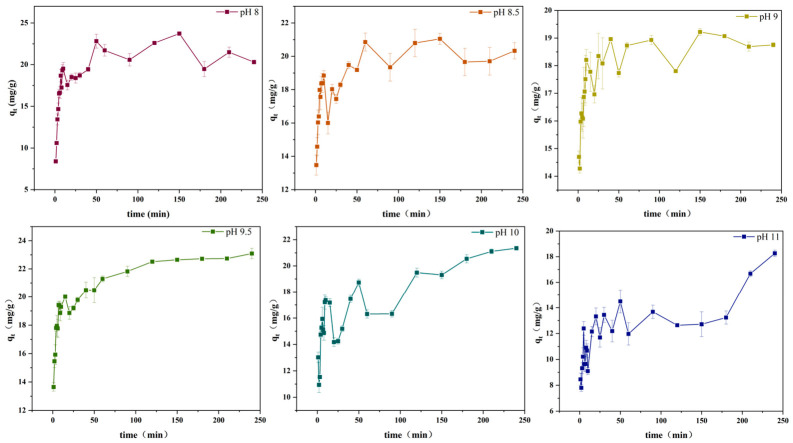
Effect of pH values on kinetic adsorption curves (0–240 min) (q_t_: adsorbed anthocyanin per gram of calcium carbonate at time t).

As shown in Figure 1, within 30 min of stirring, the maximum adsorption capacity (q_t_) first decreased and then increased with increasing pH, reaching its peak value of 20.1 mg/g at pH 9.5, indicating that this pH condition yielded the best adsorption performance. When pH ≥ 10, the adsorption capacity decreased, suggesting that the interaction between C3G and the calcium carbonate surface was significantly influenced by the initial pH. As shown by the adsorption kinetic curves, within the preset time range (1–240 min), the adsorption capacity increased rapidly to a maximum, then decreased slowly, and finally reached an equilibrium state with slight fluctuations around this value. This phenomenon could be attributed to the fact that pH affects the functional groups, charge amount, and ionic forms of the adsorbent, thereby influencing the adsorption capacity of CaCO_3_ [26].

When the initial pH is ≥10, the adsorption capacity continues to show a sustained slow increase within 240 min, indicating that the adsorption process has not yet fully reached equilibrium. This phenomenon can be attributed to the presence of multiple adsorption sites with uneven energy distribution on the calcium carbonate surface. As time progresses, sites with lower binding energy and higher mass transfer resistance are gradually occupied, allowing the adsorption process to continue over an extended period. Meanwhile, with the ongoing precipitation of calcium carbonate, the newly exposed crystal surfaces also provide additional adsorption sites, further contributing to the gradual increase in adsorption capacity. Although C3G is susceptible to some degradation under alkaline conditions, the buffering effect of rapid calcium carbonate precipitation on the system pH ensures that adsorption/coprecipitation remains the dominant mechanism within the 240 min period.

### 3.2. Determination of Characterization Indices of Lake Pigment Microparticles

As shown in Table 1, the average particle size of CaCO_3_ was 14.49 μm, while that of the C3G-CaCO_3_ composite decreased significantly to 7.84 μm, indicating a strong correlation between the addition of C3G and the reduction in calcium carbonate particle size. One possible explanation for this phenomenon is that C3G molecules may adsorb onto the surface of CaCO_3_ crystals during crystal growth, thereby interfering with the ordered deposition of Ca^2+^ and CO_3_^2−^ ions [27,28].

The specific surface area measurements showed that CaCO_3_ microparticles had a value of 3.87 m^2^/g, while MA-CaCO_3_ and C3G-CaCO_3_ exhibited values of 2.17 m^2^/g and 1.32 m^2^/g, respectively, showing a decreasing trend. Although C3G-CaCO_3_ had the smallest particle size, its specific surface area did not increase accordingly, which may be attributed to changes in pore structure and surface roughness induced by the incorporation of C3G [27].

The surface charge of calcium carbonate particles exhibits a strong pH dependence, which is related to the presence of surface -Ca^2+^ sites and their possible hydrolysis products [29]. These species can interact with ions in solution, thereby determining the net surface potential. In aqueous media, these surface sites can adsorb anions or coordinate with surrounding species, thus affecting the Zeta potential and colloidal stability of CaCO_3_ particles. In this study, both lake pigments exhibited negative surface charge characteristics. This behavior may be attributed to the adsorption of C3G molecules onto the CaCO_3_ surface. Under the experimental conditions, due to the presence of phenolic hydroxyl groups [30], C3G may exist partially in a deprotonated form, enabling electrostatic attraction or coordination interactions with surface Ca^2+^ sites. In addition, Ca^2+^-mediated bridging may also facilitate the adsorption of C3G onto the particle surface, thereby leading to a shift in surface charge.

### 3.3. Scanning Electron Microscopy (SEM) Analysis

**Figure 2:**
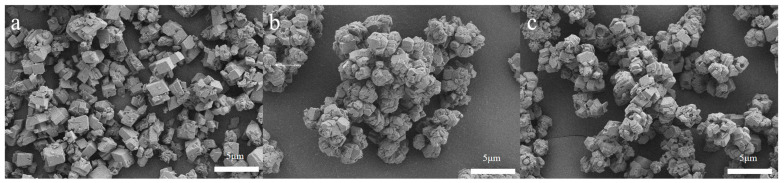
SEM images of CaCO_3_ and lake pigments ((**a**) CaCO_3_, (**b**) MA-CaCO_3_, (**c**) C3G-CaCO_3_).

As shown in Figure 2, the surfaces of the CaCO_3_ microparticles were smooth and stacked in the shape of calcite rhombohedra. The surfaces of the lake pigment microparticles showed many tiny protrusions and depressions, with rough and irregular overall morphology. This change may be due to the interaction between C3G and the surface of CaCO_3_, which affected the growth and morphology of CaCO_3_ crystals. There are Ca^2+^ sites and their possible hydrolysis products, such as Ca(OH)^2+^, on the surface of CaCO_3_, and the two are adsorbed together through electrostatic interaction. This adsorption changes the charge distribution on the surface of CaCO_3_, thereby affecting the rate and direction of crystal growth [31].

### 3.4. Crystal Form Analysis of Lake Pigments

**Figure 3:**
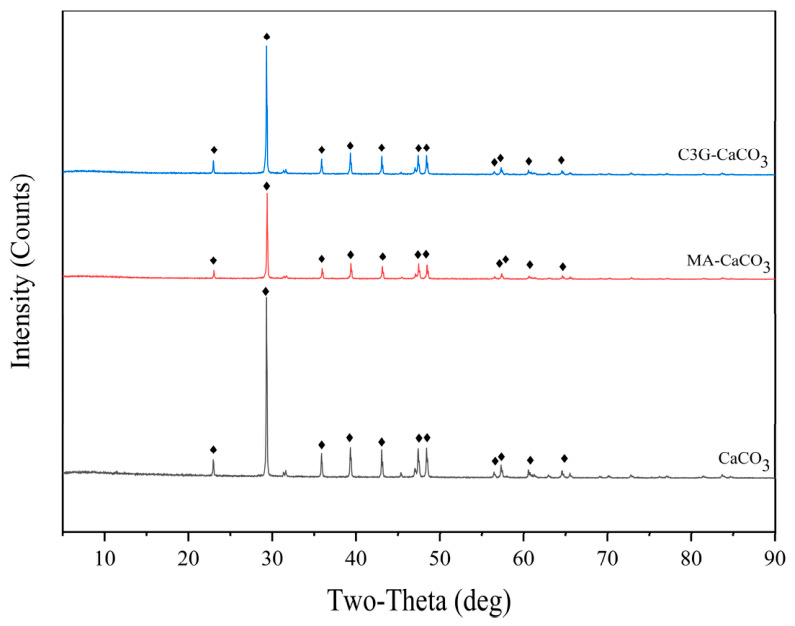
XRD patterns of CaCO_3_, MA-CaCO_3_, and C3G-CaCO_3_ microparticles.

Figure 3 shows the XRD patterns of CaCO_3_ and lake pigment microparticles. It can be seen from the figure that the XRD patterns of CaCO_3_ and lake pigments all showed a series of characteristic diffraction peaks, which were highly consistent in position, intensity, and shape, indicating that they had the same crystal structure. The experimental data were analyzed and processed by MDI Jade 6.5 software and compared with the standard card, confirming that the crystal structure was calcite. It can be concluded that C3G did not change the crystal form of CaCO_3_ in the lake pigments.

### 3.5. Adsorption Isotherm Curves

Adsorption processes can be roughly divided into physical and chemical adsorption [32]. Physical adsorption is based on weak intermolecular forces (including van der Waals forces, electrostatic interactions, hydrogen bonding, and hydrophobic effects), is reversible, and allows adsorbed molecules to migrate freely at the interface, forming monolayer or multilayer structures. Chemical adsorption, in contrast, occurs through chemical bonding, exhibiting exothermicity and selectivity. Under certain conditions, the two types of adsorption can coexist.

**Figure 4:**
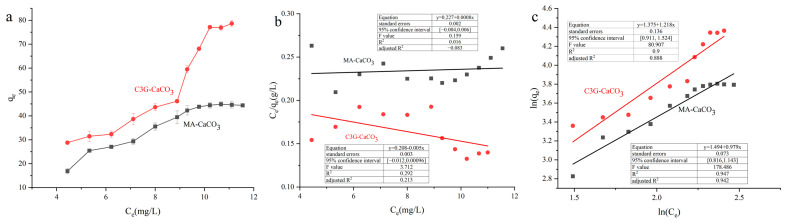
(**a**) Adsorption isotherms of MA-CaCO_3_ and C3G-CaCO_3_; (**b**) fitting of linear Langmuir equation; (**c**) fitting of linear Freundlich equation.

Figure 4 shows the isothermal adsorption data of the adsorption reaction between the two pigments and CaCO_3_ (Figure 4a), as well as the fitting results after conversion by the linear Langmuir and the Freundlich equations (Figure 4b,c). As shown in Figure 4a, with the increase in C3G concentration, the adsorption capacity q_e_ increased rapidly, and tended to be stable when C_e_ (equilibrium concentration) reached 11.11 mg/L, with a maximum adsorption capacity of about 78.63 mg/g. In contrast, the adsorption capacity q_e_ of MA at the same C_e_ concentration was lower than that of C3G, and the adsorption isotherm curve showed a slow rising trend, finally stabilizing at 45.2 mg/g. Based on these findings, we hypothesize that MA may contain additional components that compete with C3G for the limited binding sites on the CaCO_3_ surface, thereby reducing the adsorption efficiency of C3G.

The fitted parameters indicate that the determination coefficients (R^2^) of the Freundlich model are all higher than those of the Langmuir model. This suggests that the adsorption behavior of MA/C3G on calcium carbonate is more consistent with the Freundlich isotherm model. The Freundlich model parameters K_F_ and n represent the adsorption capacity and adsorption intensity, respectively. Among them, the K_F1_ and n_1_ values for C3G-CaCO_3_ were 3.96 and 1.08, respectively, while the K_F2_ and n_2_ values for MA-CaCO_3_ were 4.38 and 1.01, respectively. Therefore, based on the current isotherm fitting results, it can be preliminarily inferred that the adsorption process is predominantly governed by physical adsorption. The specific adsorption mechanism still needs to be further determined in combination with subsequent spectroscopic results. CaCO_3_ possesses a usable specific surface area and surface active sites [33], which can provide an adsorption environment for C3G.

### 3.6. XPS Analysis

**Figure 5:**
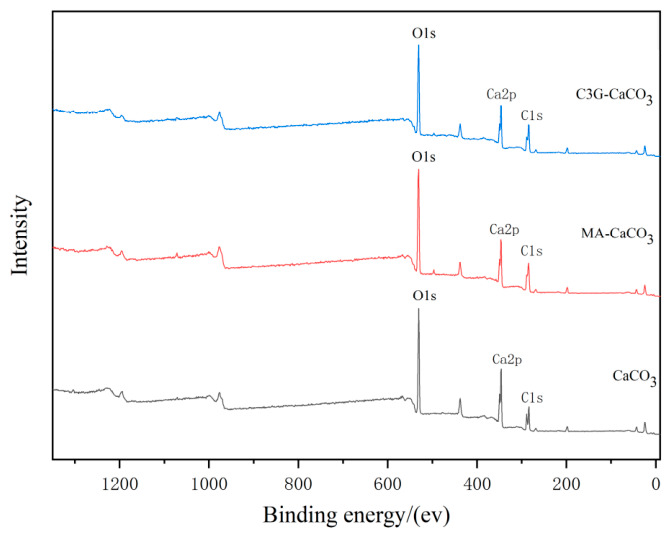
XPS spectra of CaCO_3_, MA-CaCO_3_, and C3G-CaCO_3_.

As shown in Figure 5, XPS analysis of CaCO_3_, MA-CaCO_3_, and C3G-CaCO_3_ lake pigments showed that no new elemental peaks appeared in the spectra of the lake pigments, indicating that no new elements were introduced into the lake pigments. In the XPS spectrum of CaCO_3_, Ca 2p, C 1s, and O 1s are the main characteristic peaks [34]. Among them, the binding energy of Ca 2p is usually about 347 eV, that of C 1s is about 284.8 eV, and that of O 1s is about 530 eV. The position and intensity of these characteristic peaks are the key basis for identifying CaCO_3_.

**Figure 6:**
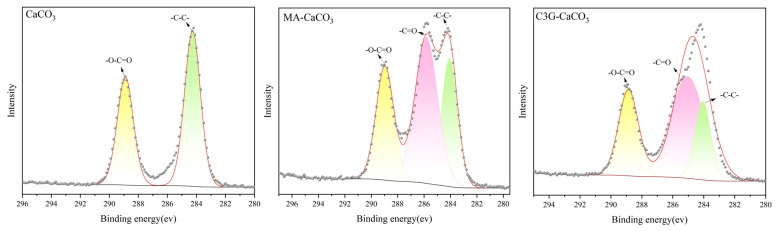
High-resolution XPS spectra of the C 1s region for CaCO_3_, MA-CaCO_3_, and C3G-CaCO_3_.

To further distinguish the chemical states of carbon, high-resolution narrow-scan analyses were performed on the C 1s regions of the three samples (Figure 6), and the binding energies of the C, O, and Ca components are shown in Table 2. As shown in Figure 6, the high-resolution C 1s spectrum of pristine CaCO_3_ can be deconvoluted into two sub-peaks. The lower binding energy component at 284.27 eV is assigned to alkyl carbon (C–C), while the higher binding energy component at 288.90 eV is attributed to carbonate carbon (CO_3_^2−^). In contrast, the high-resolution C 1s spectra of MA-CaCO_3_ and C3G-CaCO_3_ exhibit an additional sub-peak at approximately 285 eV, which can be assigned to oxygen-bonded carbon species (C=O), originating from the glycosidic/phenolic hydroxyl structures of the C3G molecule.

The appearance of the oxygen-bonded carbon species (C=O) may suggest the presence of C3G on the calcium carbonate surface. However, XPS results alone are insufficient to conclusively prove successful adsorption, and further verification through other characterization techniques is needed. In addition, the absence of new peaks or shifts in the C 1s peak of calcium carbonate indicates that the adsorption process did not cause any significant change in the surface chemical state of calcium carbonate.

### 3.7. Energy-Dispersive Spectroscopy (EDS) Analysis

**Figure 7:**
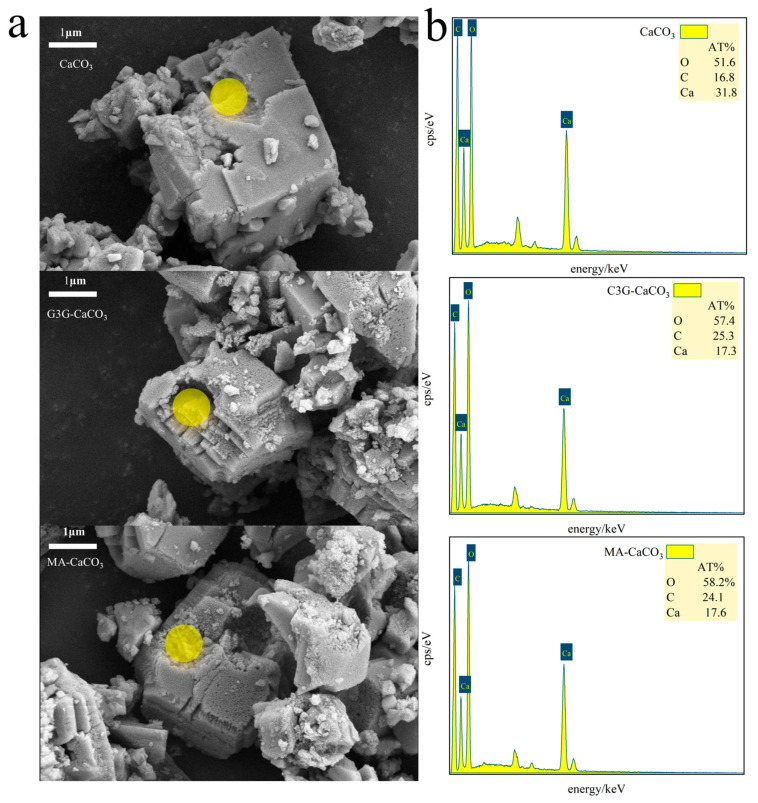
(**a**): SEM images of CaCO_3_, MA-CaCO_3_, and C3G-CaCO_3_ particles and EDS analysis scanning locations (highlighted areas). (**b**): EDS analysis spectra of cross sections of CaCO_3_, MA-CaCO_3_, and C3G-CaCO_3_ particles.

Figure 7a shows the SEM image of the lake pigment particles after liquid nitrogen brittle fracture, from which obvious cross sections can be seen in the particles. The point scanning mode was used to scan the C, O, and Ca elements on the obtained cross sections. The EDS results are shown in Figure 7b. The O element contents of CaCO_3_, MA-CaCO_3_, and C3G-CaCO_3_ were 51.6%, 58.2%, and 57.4%, respectively; the C element contents were 16.8%, 24.1%, and 25.3%, respectively; and the Ca element contents were 31.8%, 17.6%, and 17.3%, respectively. The oxygen element ratio of CaCO_3_ to C3G was 3:11, and the carbon element ratio was 1:21. The oxygen and carbon contents of the lake pigments were higher than those of CaCO_3_; the initial indication suggests that C3G may bind with calcium carbonate.

### 3.8. Fourier Transform Infrared Spectroscopy (FTIR) Analysis

**Figure 8:**
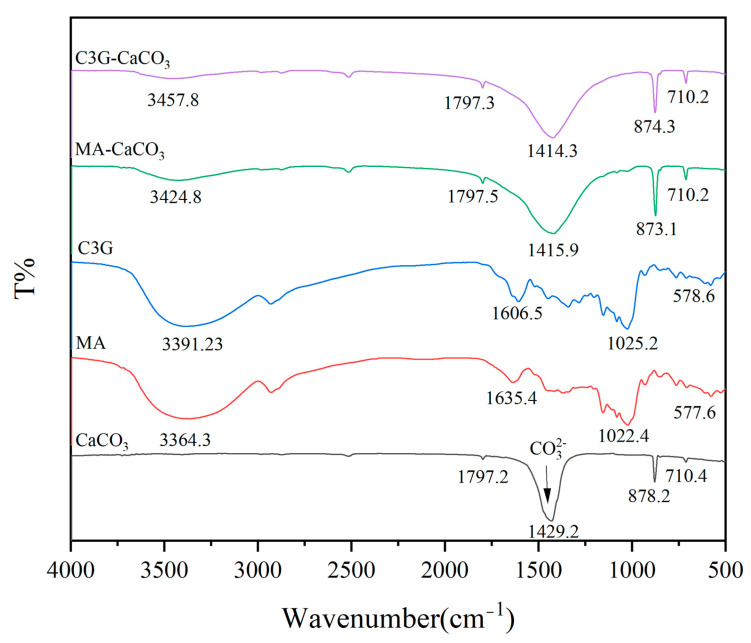
FTIR spectra of CaCO_3_, MA, C3G, MA-CaCO_3_, and C3G-CaCO_3._

Figure 8 shows the characteristic peaks of the FTIR spectra of CaCO_3_, MA, C3G, MA-CaCO_3_, and C3G-CaCO_3_. The FTIR spectra of MA-CaCO_3_ and CaCO_3_ were similar in the number and position of peaks with no obvious differences, indicating that MA and C3G had similar effects on CaCO_3_.

The FTIR spectra of C3G and MA were extremely similar, indicating that the main component of MA was C3G, and the peak shift was caused by impurity components. MA-CaCO_3_ and C3G-CaCO_3_ showed absorption peaks near 3425 cm^−1^ and 3458 cm^−1^ that did not exist in the CaCO_3_ spectrum, and these absorption peaks were derived from the characteristic peak of C3G near 3400 cm^−1^. C3G molecular structure contains groups such as hydroxyl (-OH), which usually show strong absorption peaks in the infrared spectrum, especially in the region around 3400 cm^−1^ [35] (Table 3). The detection of the -OH characteristic peak in the FTIR analysis of the lake pigments indicated that the pigment molecules bound to the CaCO_3_ particles under the existing adsorption conditions had reached the detection limit of Fourier transform infrared spectroscopy (usually 0.1–1 wt%) and could be detected, which proved the effectiveness of the method. Comparative analysis revealed that the sharp characteristic absorption band of pristine CaCO_3_ at 878.2 cm^−1^ is assigned to the out-of-plane bending vibration of carbonate (CO_3_^2−^) groups in the calcite phase. In both MA-CaCO_3_ and C3G-CaCO_3_ pigments, this band exhibited a significant decrease in intensity, along with a slight red-shift to 874.3 cm^−1^ and 873.1 cm^−1^, respectively. The observed reduction in peak intensity can be attributed to the partial coverage of the CaCO_3_ surface by adsorbed C3G/MA molecules, which reduces the accessible vibrational sites and disturbs the surface crystallographic order, as further supported by SEM and particle size analysis. Meanwhile, the red-shift toward lower wavenumbers indicates that the local vibrational environment of CO_3_^2−^ groups is altered after interaction with anthocyanins, particularly through hydrogen bonding between phenolic hydroxyl groups and surface oxygen atoms of CO_3_^2−^, as well as possible local lattice distortions induced by surface adsorption. These interactions weaken the effective force constant of the CO_3_^2−^ vibration, ultimately leading to a shift toward lower wavenumbers.

**Table 3:** Assignment of FTIR spectral peaks for CaCO_3_, MA and C3G.

Wavenumber (cm^−1^)		Peak Assignments
CaCO_3_	1429.19	The stretching vibration of CO_3_^2−^
	878.21	The out-of-plane bending vibration of CO_3_^2−^
	3364.34	The stretching vibration of the O-H group
MA	1635.39	The aromatic ring C=C skeletal stretching vibration
	1025.21	The stretching vibration of C-O in the sugar group
	3391.23	The stretching vibration of the -OH of aromatic rings and glycosyl groups
C3G	1606.49	The aromatic ring C=C skeletal stretching vibration
	1025.21	The stretching vibration of C-O in the sugar group

### 3.9. Raman Spectroscopy Analysis

**Figure 9:**
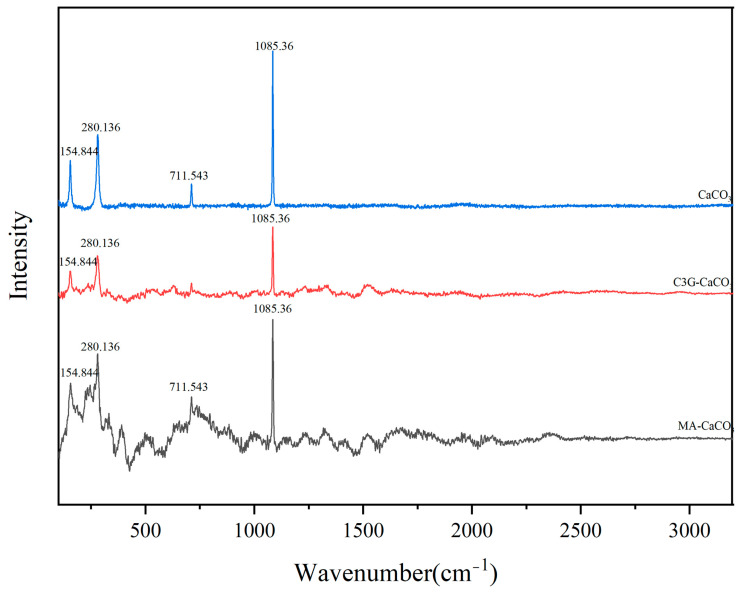
Raman spectra of CaCO_3_, MA-CaCO_3_, and C3G-CaCO_3._

In Raman spectroscopy, the Raman peaks of carbonate minerals have specific vibration modes related to the vibration of carbonate ions (CO_3_^2−^) [36]. In the Raman spectrum of CaCO_3_, the symmetric stretching vibration (ν_1_) of carbonate produces a strong stretching vibration near 1100 cm^−1^, and the stretching vibration near 700 cm^−1^ is caused by the in-plane bending vibration (ν_4_) [37]. As shown in Figure 9, the most prominent peak at 1086 cm^−1^ corresponds to the (CO_3_^2−^) ν_1_ symmetric stretching vibration, and the peak at 712 cm^−1^ corresponds to the in-plane bending vibration (ν_4_). The absorption bands at 710 cm^−1^ and 282 cm^−1^ are typical calcite Raman peaks [38]. MA and C3G affected the response values of CaCO_3_ peaks at 711.543 cm^−1^, 280.136 cm^−1^ and 154.844 cm^−1^, but did not cause peak shift, indicating that under the employed Raman detection conditions, no clear evidence of new covalent bond formation was observed. This indicates that MA and C3G may interact with CaCO_3_ through physical means rather than the formation of chemical bonds.

### 3.10. Thermogravimetric Analysis (TGA) and Differential Scanning Calorimetry (DSC)

**Figure 10:**
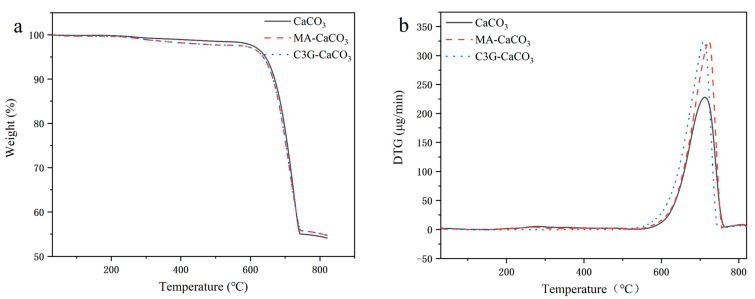
(**a**): TGA curves of CaCO_3_, MA-CaCO_3_, and C3G-CaCO_3_; (**b**): DTG curves of CaCO_3_, MA CaCO_3_, and C3G-CaCO_3._

As shown in Figure 10a, the thermogravimetric curves of CaCO_3_, MA-CaCO_3_, and C3G-CaCO_3_ showed roughly the same trend. The thermal degradation process of CaCO_3_, MA-CaCO_3_, and C3G-CaCO_3_ can be divided into two parts: the first part is the water loss process, which continues up to about 600 °C, with a slight and gentle decrease in the TG curve, mainly attributable to the removal of adsorbed water and/or residual volatile components. The second part is the pyrolysis part, from about 600 °C to 800 °C, where CaCO_3_ decomposes to form CaO and CO_2_, and CO_2_ escapes in the gaseous form, resulting in a significant decrease in sample mass [39]. The residual masses of CaCO_3_ and lake pigments at 800 °C were 54.50%, 55.14%, and 55.13%. Based on the decomposition reaction, the theoretical residual mass fraction of CaO after complete decomposition of CaCO_3_ is calculated to be 56.03%. The slight deviation between the experimental residual mass and the theoretical value may be attributed to incomplete decomposition of CaCO_3_ under the employed TGA conditions, as well as kinetic limitations associated with CO_2_ diffusion during thermal decomposition. In addition, for the MA-CaCO_3_ and C3G-CaCO_3_ samples, the presence of organic surface species may slightly influence the thermal decomposition behavior and lead to minor changes in residual mass due to trace carbonaceous residues remaining after heat treatment. As shown in Figure 10b, the initial decomposition temperatures of CaCO_3_, MA-CaCO_3_, and C3G-CaCO_3_ were determined to be approximately 600 °C, 590 °C, and 570 °C, respectively. Meanwhile, the maximum decomposition rate temperature (T_max_, corresponding to the DTG peak) of pristine CaCO_3_ was located at 712 °C. After hybridization with anthocyanins, the T_max_ of MA-CaCO_3_ and C3GCaCO_3_ shifted slightly to 720 °C and 710 °C, respectively, indicating that the incorporation of C3G altered the thermal decomposition behavior of CaCO_3_ and significantly affected its decomposition kinetics.

**Figure 11:**
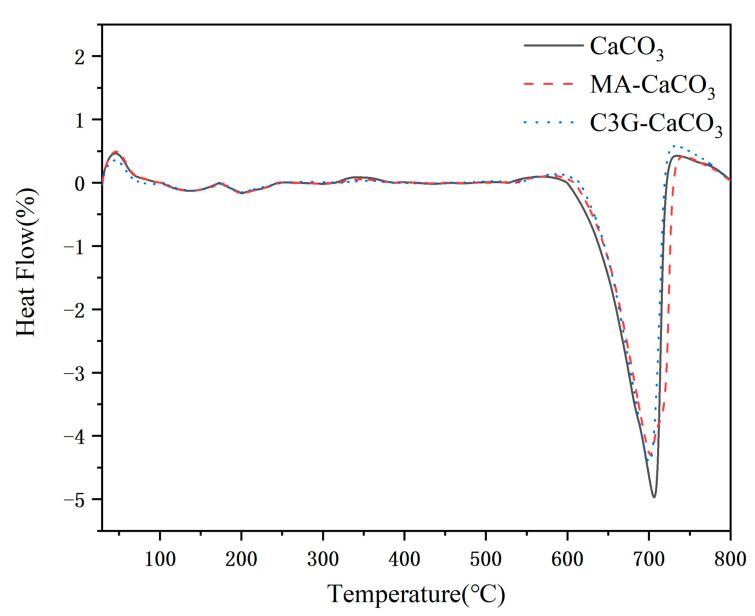
DSC curves of CaCO_3_, MA-CaCO_3_, and C3G-CaCO_3._

DSC technology can accurately determine the heat absorbed or released by the sample during heating. As shown in Figure 11, the DSC curves of CaCO_3_, MA-CaCO_3_, and C3G-CaCO_3_ showed similar trends, all with an obvious endothermic peak at about 700 °C, which was attributed to the endothermic process of light CaCO_3_ decomposition to produce carbon dioxide (CO_2_). However, compared with pure CaCO_3_, the endothermic peak intensities of MA-CaCO_3_ and C3G-CaCO_3_ were different, indicating that the introduction of pigments may affect the thermal stability of CaCO_3_ to a certain extent, thus changing its thermal decomposition behavior.

## 4. Conclusions

In this study, cyanidin-3-O-glucoside (C3G) was used as the target pigment and calcium carbonate (CaCO_3_) as the matrix to prepare MA-CaCO_3_ and C3G-CaCO_3_ lake pigments via the coprecipitation method. The regulatory law of pH on the MA adsorption process by CaCO_3_ was clarified, and the interaction characteristics between CaCO_3_ and the two pigments, as well as the formation mechanism of the lake pigments, were systematically investigated. The results showed that pH was a key factor affecting the adsorption of MA by CaCO_3_, with the optimal adsorption effect achieved at pH 9.5. The introduction of C3G reduced the particle size of CaCO_3_, reversed its surface charge from positive to negative, and led to a decreasing trend in specific surface area. In terms of micromorphology, pure CaCO_3_ presented as smooth calcite rhombohedrons, while the lake pigments had rough and irregular surfaces, with their crystal form remaining unchanged as calcite. The adsorption of MA and C3G on the surface of CaCO_3_ conformed to the Freundlich isotherm model. Meanwhile, the comparison of adsorption performance indicated that C3G had higher binding efficiency with CaCO_3_, and the C3G-CaCO_3_ lake pigment exhibited higher maximum adsorption capacity. XPS and EDS analyses indicated that C3G was clearly associated with calcium carbonate, yet no characteristic features of new chemical bond formation were observed in the spectral data. Combined with microscopic morphological observations and the adsorption experimental results, it is inferred that C3G is primarily dominated by surface adsorption, while the possibility that a portion of C3G may be encapsulated within the calcium carbonate particles cannot be ruled out. However, this inference still requires further verification. FTIR and Raman spectroscopy further corroborated the above conclusion that no chemical bond cleavage or formation occurred. TGA-DSC results indicated that the introduction of C3G altered its thermal decomposition behavior, while the overall thermal degradation trend remained unchanged. In summary, the CaCO_3_-based lake pigment system exhibits good performance in terms of pigment adsorption efficiency, and given the use of food-grade CaCO_3_ as the matrix, it holds potential for further exploration in edible applications. This study provides a theoretical basis and technical reference for the adsorption-based strategy of natural pigments, and broadens the perspective for subsequent in-depth research on functional applications.

## Figures and Tables

**Table 1:** Basic characteristics of CaCO_3_, MA−CaCO_3_, and C3G−CaCO_3_ microparticles.

	Particle Size (μm)	Zeta Potential (mV)	Specific Surface Area (m^2^/g)
CaCO_3_	14.49 ± 0.01 ^a^	8.08 ± 0.29 ^a^	3.87 ± 0.25 ^a^
MA-CaCO_3_	10.41 ± 0.09 ^b^	−7.44 ± 0.35 ^c^	2.17 ± 0.16 ^b^
C3G-CaCO_3_	7.84 ± 0.03 ^c^	−4.97 ± 0.32 ^b^	1.32 ± 0.01 ^c^

All experiments were performed in three independent replicates. Values not sharing the same superscript letters in one column are significantly different (*p* < 0.05).

**Table 2:** Surface composition and binding states of Ca, C, and O elements in CaCO_3_, MA-CaCO_3_, and C3G-CaCO_3._

	CaCO_3_		MA-CaCO_3_		C3G-CaCO_3_	
	Binding Energy (eV)	Atomic Percentage (%)	Binding Energy (eV)	Atomic Percentage (%)	Binding Energy (eV)	Atomic Percentage (%)
	284.27	10.64	284.07	5.75	284.06	4.40
C 1s	-	-	285.83	9.32	285.05	12.71
	288.90	7.47	288.97	5.62	288.90	5.88
O 1s	530.79	19.44	531.04	21.42	530.84	18.17
Ca 2p	346.45	4.88	346.56	3.35	346.43	3.64
	350	2.34	350.10	1.58	349.98	1.7

## Data Availability

The data presented in this study are available on request from the corresponding author. The data are not publicly available due to privacy restrictions.

## Supplementary Materials

The following supporting information can be downloaded at https://www.mdpi.com/article/10.3390/foods15132409/s1: Figure S1: Chromatograms of C3G separation and identification (a: preparative HPLC chromatogram of C3G; b: analytical HPLC chromatogram of C3G). Figure S2: Mass spectra of standard and extract (a: standard; b: extract).
