# Supplementary material for: Preparation of Lake Pigment from Calcium Carbonate and Cyanidin-3-O-Glucoside: Structural Characterization and Formation Mechanism
Source: Foods. 2026 Jul 7;15(13):2409. doi: 10.3390/foods15132409 (PMC13360927; doi:10.3390/foods15132409)
Supplement: Supplementary file 1 [file foods-15-02409-s001.zip › foods-4362240-supplementary.pdf]

（1）Materials and Methods

The isolated high-purity C3G solid powder and the C3G standard were subjected to comparative analysis by liquid chromatography and mass spectrometry. The liquid chromatography and mass spectrometry conditions were as follows:

Liquid chromatography conditions: Chromatographic separation was performed on an ACQUITY UPLC CSH column (2.1 × 50 mm, 1.7 μm). Acetonitrile was used as mobile phase A, and water containing 0.1% formic acid was used as mobile phase B. The flow rate was 0.3 mL/min, the column temperature was maintained at 40 °C, and the detection wavelength was 530 nm. The elution program was as follows: 0–1 min, hold at 5% A and 95% B; 1–6 min, A increased linearly from 5% to 95% and B decreased correspondingly from 95% to 5%.

Mass spectrometry conditions: Mass spectrometry was performed using an electrospray ionization (ESI) source in positive ion mode. The ion source temperature was set to 500 °C, the capillary voltage was 2.5 kV, and the cone voltage was 40 V. The desolvation gas flow rate was 900 L/h. The mass-to-charge ratio (m/z) was scanned in the range of 60–1000.

（2）Results

Refer to the elution parameter specifications for analytical liquid chromatography, multiply the mobile phase flow rate by a scaling factor of 10 to determine the initial separation conditions for preparative liquid chromatography. Adjust the polarity and flow rate of the mobile phase based on the separation performance and resolution time of the pigment components, ultimately establishing the optimal elution conditions.

As shown in Figure , at a wavelength of 530 nm and a retention time of 7.588 min, a distinct peak corresponding to the target component appeared, exhibiting a red color; the sample was collected and dried. Although preparative HPLC demonstrates high separation efficiency, the dried pigment monomer was subjected to HPLC analysis for further identification. The results revealed that the separated compound exhibited a well-defined peak on the analytical HPLC chromatogram with a retention time of 2.34 min.

Figure b shows the liquid chromatographic profiles of the C3G standard sample and the isolated C3G sample. Under identical chromatographic conditions, the peak elution time for the standard sample was 2.54 min, while that for the isolated sample was 2.34 min, indicating a minimal difference in retention times and confirming they represent the same compound. However, the profile exhibits additional peaks, necessitating further identification through analysis of molecular ion peaks in mass spectrometry.

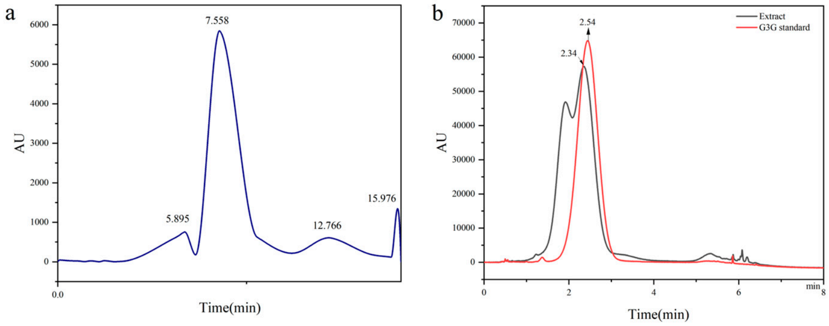

Figure S1. Chromatograms of C3G separation and identification (a: Preparative HPLC chromatogram of C3G; b: Analytical HPLC chromatogram of C3G).

As shown in **Figure** a, the molecular ion peak of the C3G standard sample appears at m/z 449, with another prominent peak at m/z 287. It can be inferred that the peak at m/z 449 corresponds to the C3G molecule, which yields chrysanthemisin upon loss of a glucose residue, as evidenced by the ion peak at m/z 287. The mass spectrum in **Figure** b closely resembles that in **Figure** a, confirming that the purified sample primarily consists of C3G.

In summary, based on data such as retention time, λmax, molecular ion peaks, and main fragmentation patterns, the purified pigment is nearly identical to the reference standard, further confirming its identity as C3G.

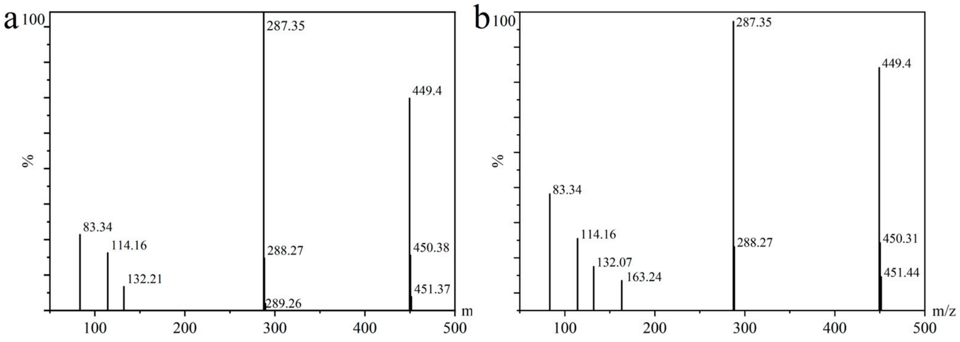

Figure S2. Mass spectra of standard and extract (a: Standard; b: Extract).
